# Long-term survival in patients with isolated pulmonary valve stenosis: a not so benign disease?

**DOI:** 10.1136/openhrt-2021-001836

**Published:** 2021-09-14

**Authors:** Kristofer Skoglund, Annika Rosengren, Georgios Lappas, Maria Fedchenko, Zacharias Mandalenakis

**Affiliations:** 1 Department of Molecular and Clinical Medicine, University of Gothenburg, Goteborg, Sweden; 2 Department of Cardiology, Sahlgrenska University Hospital, Goteborg, Sweden

**Keywords:** pulmonary valve stenosis, congenital heart disease, epidemiology

## Abstract

**Background and objectives:**

During the last decades, the survival rates in patients with congenital heart disease have increased dramatically, particularly in patients with complex heart malformations. However, the survival in patients with simple defects is still unknown. We aimed to determine the characteristics and the risk of mortality in patients with isolated pulmonary valve stenosis (PS).

**Methods:**

Swedish inpatient, outpatient and cause of death registries were used to identify patients born between 1970 and 2017 with a diagnosis of PS, without any other concomitant congenital heart lesion. For each patient with PS, 10 control individuals without congenital heart disease were matched by birth year and sex from the total population registry. We used median-unbiased method and Kaplan-Meier survival analysis to examine the risk of mortality.

**Results:**

We included 3910 patients with PS and 38 770 matched controls. The median age of diagnosis of PS was 0.7 years (IQR 0.3–7.0). During a median follow-up of 13.5 years (IQR 6.5–23.5), 88 patients with PS and 192 controls died; 500 patients with PS (12%) underwent at least one transcatheter or surgical valve intervention. The overall mortality rate was significantly higher in patients with PS compared with matched controls (HR 4.67, 95% CI 3.61 to 5.99, p=0.001). Patients with an early diagnosis of PS (0–1 year) had the highest risk of mortality (HR 10.99, 95% CI 7.84 to 15.45).

**Conclusions:**

In this nationwide, register-based cohort study, we found that the risk of mortality in patients with PS is almost five times higher compared with matched controls. Patients with an early diagnosis of PS appears to be the most vulnerable group and the regular follow-up in tertiary congenital heart units may be the key to prevention.

**Key questions**

**What is already known about this subject?**

- During the last decades survival in patients with congenital heart disease has increased dramatically particularly in complex heart malformations. Isolated pulmonary valve stenosis (PS) has in mild forms been considered associated with normal survival.

**What does this study add?**

- We found that the mortality risk for patients with PS was five times higher than in matched controls for both operated an unoperated patients. The highest risk was observed in patients with a diagnosis of PS in the first year of life appearing at an HR of 10.99.

**How might this impact on clinical practice?**

- In this national cohort study, we found that patients with PS had markedly higher mortality risk than matched controls and diagnosis of PS can not be considered a benign disease. Patients with diagnosis of PS in early life appears as a high-risk group and should be paid close attention in long-term follow-up.

## Introduction

Evolution of care for patients with congenital heart disease has led to more than 97% of patients currently reaching adulthood.^1–3^ Isolated pulmonary valve stenosis (PS) is a relatively common malformation occurring in 8%–10% of all live births with congenital heart disease corresponding to one in every thousand babies born.^4 5^ Treatment for PS was originally limited to surgical valvulotomy, but in recent decades, percutaneous balloon valvuloplasty has become the preferred treatment.^6 7^ Long-term, event-free survival after valve intervention is >90%, but complications after interventions for PS include pulmonary valve regurgitation with possible right ventricular volume overload, occurs in one-third of patients.^8–11^ Furthermore right ventricular hypertrophy and stiffness related to right ventricular alterations can cause impaired function.^12^ In clinical study cohorts, untreated mild PS has been considered to be a benign disease and may associated with normal survival as well as a low risk for progression of this valvular disease^13–17^ Population-based studies with long-term follow-up in patients with PS with and without cardiac intervention are, however, limited and we are aware of no study that have followed unselected patients with PS in an entire nation from birth.

Therefore, this study aimed to determine the characteristics and the risk of mortality in patients with PS on a national level using Swedish medical registries.

## Methods

### Population

The National Cause of Death Registry (complete since 1968), the National Hospital Inpatient Registry (initiated in 1964, complete since 1987, but with coverage of thoracic surgery since 1970) and the National Hospital Outpatient Registry (complete since 2001) were used to identify all patients born from 1970 to 2017 who had a registered diagnosis of PS at death, or at any visit to a Swedish hospital during a follow-up. Identification of patients with PS including subvalvular PS was by diagnosis codes according to the International Classification of Diseases, Revision 8 and Related Health Problems (ICD-8 and ICD-9: 7466, 7460, 424D, I370, I372; ICD-10: Q221, Q223 and Q243). Patients with any concomitant congenital heart lesions were excluded from the analysis, except for PS patients with also a pulmonary valve regurgitation diagnosis (ICD-8 and ICD-9: I371; ICD-10: Q22.2). For each patient with isolated PS, 10 control individuals without congenital heart disease were randomly matched by birth year and sex from the total population register.

Surgical or percutaneous valve interventions related to PS were defined as the presence of any code involving intervention to the right ventricular outflow tract and/or pulmonary valve according to classification of surgical Procedures.^18^

All personal identifiers were removed from the final dataset and replaced by a code.

### Statistical analysis

Descriptive statistics were used to present demographic data. Data are presented as mean, median or IQR, as well as absolute numbers and percentages.

Mortality rates for cases versus controls, with valve interventions versus no valve interventions and men versus women, were estimated in different strata defined by birth period and age at first known registration of the disease and compared with rate ratios. The rate ratios are estimated according to the median-unbiased method (mid-p) and the corresponding confidence according to exact methods (mid-p), (R-package ‘epitools’, Epidemiology Tools, V.0.5–10.1, Tomas J. Aragon).

Kaplan-Meier plots with pointwise 95% CIs are presented illustrating group differences along with p values from log-rank tests. A two-tailed p<0.05 was considered significant. Matching of cases and controls was performed at birth and stratified by birth year and sex.

The data preparation was performed with logic programming system SWI-Prolog V.8.3 (www.swi-prolog.org). The statistical analyses were performed using R V.4.0 (R Foundation for Statistical Computing, Viena, Austria, www.r-project.org).

### Patient and public involvement

There was no public or patient involvement in this study.

## Results

### Baseline characteristics

Table 1 shows the characteristics of the study population. We included 3910 patients with PS and 38 770 matched controls; 54.7% were female. The median age of PS diagnosis at registration was 0.7 years (IQR 0.3–7.0) and for patients with PS who underwent valve intervention was 0.5 years (IQR 0.3–3.7). A total of 2051 patients with PS (52.5%) were diagnosed within the first year of life and 500 patients with PS (12.7%) had at least one valve intervention (transcatheter or surgical).

**Table 1:** Baseline characteristics of patients with isolated PS and controls

		PS, total (n=3910)	Controls, total (n=38 770)	PS, no valve interventions (n=3410)	Controls (n=33 790)	PS, valve interventions (n=500)	Controls (n=4980)
Female, n (%)		2139 (54.7)	21 060 (54.3)	1901 (55.7)	18 700 (55.3)	238 (47.6)	2360 (47.4)
Median age at registration, years (IQR)		0.7 (0.3–7.0)	–	0.8 (0.3–7.7)	–	0.5 (0.3–3.7)	–
Median follow-up, years (IQR)		13.5 (6.5–23.5)	13.8 (6.8–24.5)	13.5 (6.5–23.5)	13.8 (6.8–24.5)	13.9 (6.6–21.5)	14.5 (6.7–21.5)
Birth period							
1970–1979	No of patients, n	246	2460	215	2150	31	310
	Median age at registration, years (IQR)	12.1 (5.3–28.9)	–	14.3 (5.3–29.4)	–	6.6 (4.5–13.3)	–
	Median follow-up, years (IQR)	42.5 (39.6–45.5)	42.5 (40.5–45.5)	42.5 (40.5–45.5)	42.5 (40.5–45.5)	41.5 (39.5–44.0)	41.5 (39.5–44.3)
1980–1989	No of patients, n	433	4330	396	3960	37	370
	Median age at registration, years (IQR)	14.6 (3.1–19.4)		14.9 (3.6–19.6)		6.8 (0.9–17.4)	
	Median follow-up, years (IQR)	31.8 (29.5–34.5)	31.9 (29.6–34.5)	31.5 (29.5–34.5)	31.8 (29.7–34.5)	34.5 (29.6–35.6)	34.5 (29.6–35.6)
1990–1999	No of patients, n	786	7860	659	6590	127	1270
	Median age at registration, years (IQR)	7.1 (1.5–12.1)		7.8 (2.5–12.4)		1.7 (0.4–6.8)	
	Median follow-up, years (IQR)	22.5 (19.8–25.5)	22.5 (20.5–25.5)	22.5 (20.5–25.5)	22.8 (20.5–25.5)	21.5 (19.6–23.8)	21.5 (19.7–23.8)
2000–2009	No of patients, n	1294	12 770	1146	11 300	148	1470
	Median age at registration, years (IQR)	0.6 (0.3–2.6)	–	0.7 (0.3–2.7)	–	0.5 (0.3–1.9)	–
	Median follow-up, years (IQR)	12.6 (10.5–15.5)	12.6 (10.5–15.5)	12.6 (10.5–15.5)	12.6 (10.5–15.5)	12.7 (10.5–15.6)	12.7 (10.5–15.6)
2010–2016	No of patients, n	1151	11 350	994	9790	157	1560
	Median age at registration, years (IQR)	0.4 (0.2–0.7)	–	0.4 (0.3–0.7)	–	0.3 (0.2–0.5)	–
	Median follow-up, years (IQR)	3.9 (2.5–5.9)	4.0 (2.6–5.9)	3.8 (2.5–5.8)	3.9 (2.5–5.8)	4.8 (3.5–6.5)	4.8 (3.5–6.5)
Age of diagnosis							
0–1 year	No of patients, n	2051	20 308	1740	17 219	311	3089
	Median age at registration, years (IQR)	0.4 (0.2–0.5)	–	0.4 (0.2–0.5)	–	0.3 (0.2–0.5)	–
	Median follow-up, years (IQR)	8.5 (3.7–13.8)	8.7 (3.9–14.6)	8.3 (3.6–13.6)	8.7 (3.8–14.5)	8.6 (4.8–15.8)	8.7 (4.8–15.9)
1–10 years	No of patients, n	1103	10 902	962	9491	141	1411
	Median age at registration, years (IQR)	3.8 (2.0–6.7)	–	3.8 (1.9–6.6)	–	3.7 (2.0–6.7)	–
	Median follow-up, years (IQR)	16.5 (10.5–22.5)	16.6 (10.5–22.5)	15.6 (9.6–22.5)	16.5 (9.7–22.5)	20.5 (12.7–26.5)	20.5 (12.5–26.5)
>10 years	No of patients, n	756	7560	708	7080	48	480
	Median age at diagnosis, years (IQR)	16.0 (12.8–21.5)	–	16.0 (12.8–21.3)	–	16.1 (12.5–22.3)	–
	Median follow-up, years (IQR)	28.5 (23.5–35.5)	28.5 (23.5–35.5)	28.5 (23.5–34.5)	28.5 (23.5–35.5)	29.5 (22.3–37.5)	29.5 (21.5–37.5)

PS, pulmonary stenosis.

### Mortality

**Figure 1:**
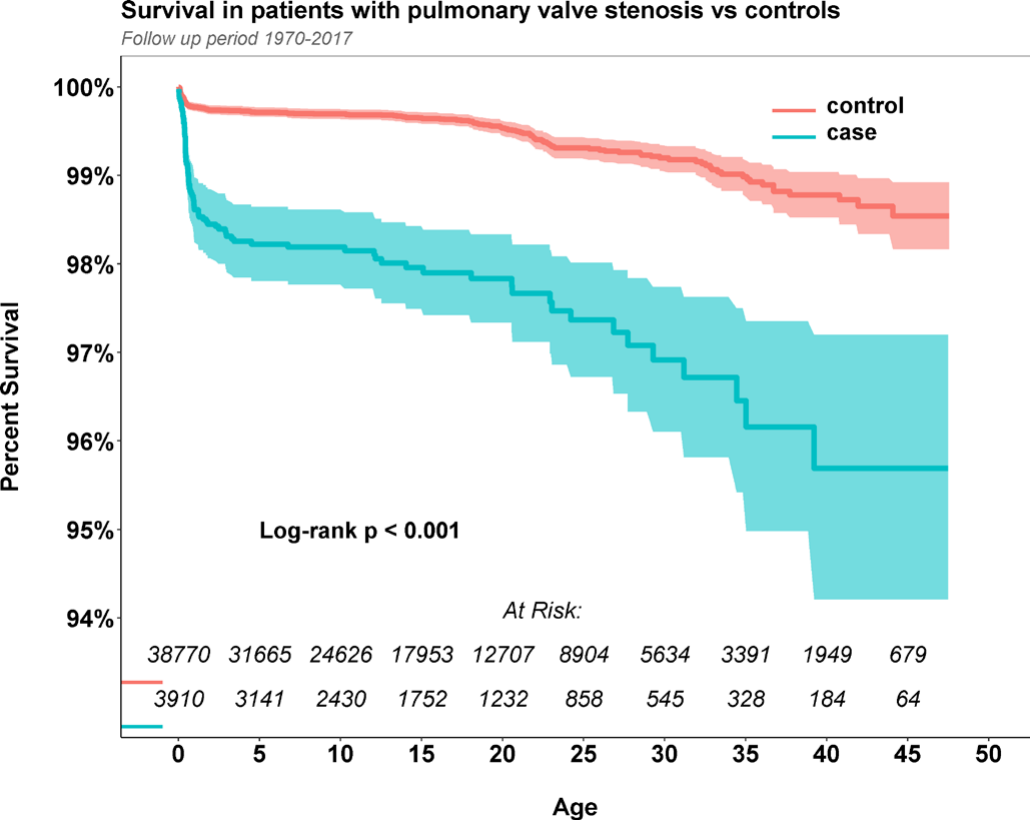
Kaplan-Meier estimated survival in patients with isolated pulmonary stenosis and matched controls.

At the end of the study period, 88 patients with PS (2.3%) and 192 controls (0.5%) died. The median follow-up time from birth was 13.5 years (IQR 6.5–23.5). Overall Kaplan-Meier estimated survival in cases and controls is shown in figure 1. The mortality was significantly higher in patients with PS than in controls (p=0.001), particularly during the first year after birth. The risk of mortality was 4.67 times higher (95% CI 3.61 to 5.99, p=0.001) in patients with PS compared with controls. Thus, the risk of mortality by birth period and age at diagnosis is shown in table 2. All of the prespecified periods of birth showed a significant effect on the risk of mortality in patients with PS compared with controls. Patients with PS diagnosis within the first year after birth, had a markedly elevated risk of mortality, 10.99 (95% CI 7.84 to 15.45, p<0.001). No excess risk was observed in patients with a PS diagnosis at the age 1 to 10 years, or >10 years.

**Table 2:** All-cause mortality according to birth cohort and to age at diagnosis in patients with isolated PS and controls

	PS, total		Controls, total		Risk of mortality	
	No of deaths among patients/total no of patients (%)		No of deaths among controls/total no of controls (%)		HR (95% CI)	P value
	Death events, n	Mortality rate per 100 000 person-years	Death events, n	Mortality rate per 100 000 person-years		
All	88/3910 (2.3)		192/38 770 (0.5)		4.67 (3.61 to 5.99)	<0.0001
Birth period						
1970–1979	15	149.2 (83.5–246.1)	39	37.3 (26.5–51.0)	4.02 (2.14 to 7.16)	<0.0001
1980–1989	23	171.0 (108.4–256.7)	48	34.5 (25.4–45.7)	4.98 (2.97 to 8.11)	<0.0001
1990–1993	24	137.2 (87.9–204.2)	46	25.8 (18.9–34.4)	5.35 (3.21 to 8.68)	<0.0001
2000–2009	14	84.9 (46.4–142.5)	33	20.1 (13.9–28.3)	4.25 (2.19 to 7.79)	<0.0001
2010–2016	12	246.9 (127.6–431.3)	26	54.0 (35.2–79.1)	4.61 (2.23 to 8.97)	<0.0001
Age at registration, years						
0–1 year	71	8944.7 (6985.9–11 282.6)	64	813.4 (626.4–1038.7)	10.99 (7.84 to 15.45)	<0.0001
1–10 years	6	123.2 (45.2–268.1)	52	107.5 (80.3–140.9)	1.17 (0.45 to 2.53)	0.751
>10 years	11	79.2 (39.5–141.7)	76	54.7 (43.1–68.5)	1.47 (0.73 to 2.64)	0.249

PS, pulmonary stenosis.;

Only 7 out of the 88 deaths occurred among the 500 patients PS that underwent valve intervention; both valve intervention and non-valve intervention group of patients with PS displayed an increased risk of mortality compared with controls; however, with a borderline significance in the valve intervention group. Among patients with PS that did not undergo valve intervention, the risk of mortality was 5.07 (95% CI 3.87 to 6.60) compared with controls. Higher mortality rates were observed in operated and unoperated patients with PS between 1990 and 2016 for patients with a PS diagnosis within the first decade of life (table 3).

**Table 3:** All-cause mortality in patients with isolated PS (with and without valve interventions) and controls, according to birth cohort, to age at diagnosis

	With valve intervention		No valve intervention	
	No of deaths among patients with PS and valve interventions/total no of valve intervened patients with PS (%)	RR for mortality (95% CI, p value)	No of deaths among patients with PS and no intervention/total no of patients with no interventions PS (%)	RR for mortality (95% CI, p value)
	7/500 (1.4)	2.47 (0.99 to 5.36, 0.029)	81/3410 (2.4)	5.07 (3.87 to 6.60,<0.0001)
	No of death events/no total (%)	RR for mortality (95% CI)	No of death events/no total (%)	RR for mortality (95% CI)
Birth period				
1970–1989	2/68 (2.9)	1.64 (0.24 to 6.02, 0.565)	36/611 (5.9)	5.07 (3.37 to 7.50,<0.0001)
1990–2016	5/432 (1.2)	3.22 (1.03 to 8.31, 0.018)	45/2799 (1.6)	5.10 (3.53 to 7.25,<0.0001)
Age at registration, years				
0–10	7/452 (1.5)	3.00 (1.18 to 6.64, 0.008)	70/2702 (2.6)	7.85 (5.73 to 10.70,<0.0001)
>10	0/48 (0.0)	–	11/708 (1.6)	1.57 (0.78 to 2.84, 0.174)

**Figure 2:**
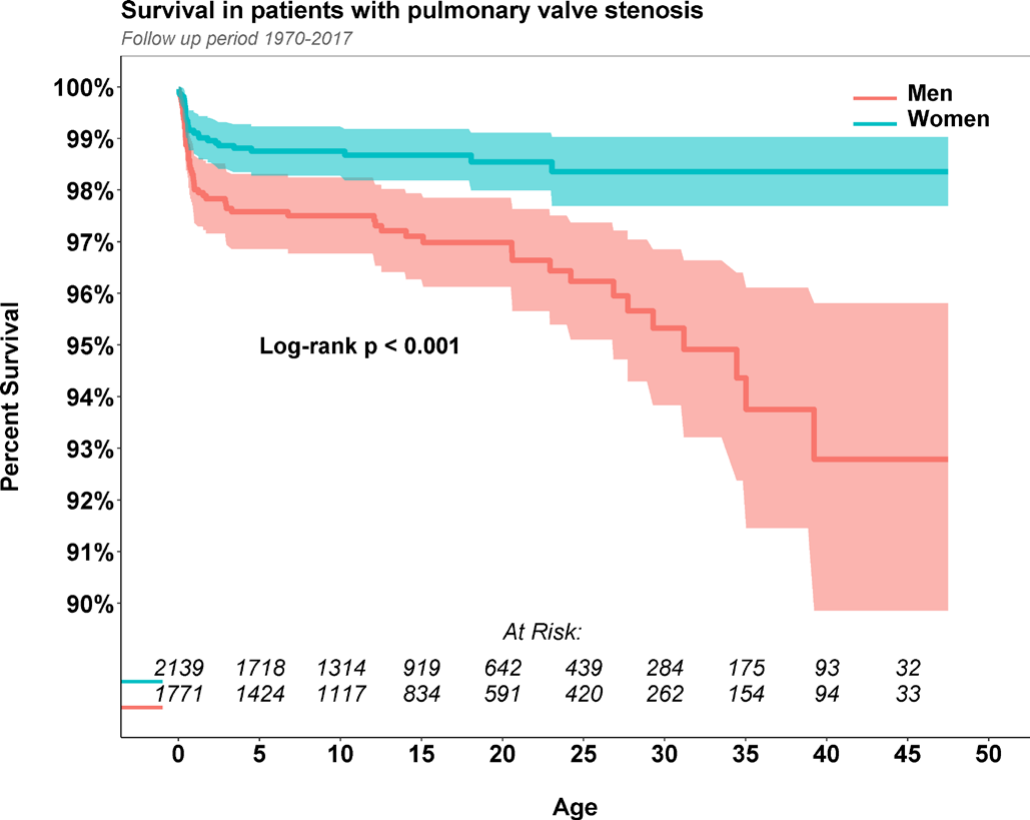
Kaplan-Meier estimated survival in patients with isolated pulmonary stenosis according to sex.

PS, pulmonalis stenosis; RR, relative risk.;

In Kaplan-Meier survival analysis of women and men, there was significantly better survival in women than in men (figure 2). Both men and women appeared to have a higher risk of mortality compared with controls (RR 5.66, 95% CI 4.09 to 7.74 and RR 3.48, 95% CI 2.24 to 5.24, both p<0.001, respectively). Furthermore, men and women with a diagnosis of PS before 10 years old appeared to have a higher risk of mortality (table 4).

**Table 4:** All-cause mortality in patients with isolated PS and controls according to sex, birth cohort and age at diagnosis

	Men		Women	
	Death events among men with PS/total no of men with PS, n (%)	HR for mortality (95% CI, p value)	Death events among women with PS/total no of women with PS, n (%)	HR for mortality (95% CI, p value)
	59/1771 (3.3)	5.66 (4.09 to 7.74, <0.0001)	29/2139 (1.4)	3.48 (2.24 to 5.24, <0.0001)
Birth period				
1970–1989	28/339 (8.3)	6.06 (3.75 to 9.57, <0.0001)	10/340 (2.9)	2.71 (1.27 to 5.25, 0.004)
1990–2016	31/1432 (2.2)	5.37 (3.43 to 8.23, <0.0001)	19/1799 (1.1)	4.13 (2.36 to 6.95, <0.0001)
Age at registration, years				
0–10	50/1427 (3.5)	8.09 (5.57 to 11.68, <0.0001)	27/1727 (1.6)	5.36 (3.31 to 8.47, <0.0001)
>10	9/344 (2.6)	2.13 (0.97 to 4.17, 0.039)	2/412 (0.5)	0.65 (0.10 to 2.13, 0.484)

PS, pulmonalis stenosis.;.

When analysing ICD codes for the leading cause of death among patients with PS, cardiac-related death was the most common cause. Cardiac-related death accounted for 42 of the 88 deaths (48%) in patients with PS. Moreover, 35 (40%) of the deaths were in PS patients with syndromes, cystic fibrosis or major malformations of inner organs or central nervous system. Mean age at death for men (n=59) were 7.1 years (median 0.8) and for women (n=29) 2.6 years (median 0.56).

## Discussion

In this nationwide, register-based cohort study, we studied all patients with PS from birth to over the last half century in Sweden. To the best of our knowledge, this is the largest population-based study of patients with PS in this field.

The main finding of our study is that the mortality in patients with PS was almost five times higher than in matched controls without congenital heart disease, with over 90% deaths occurring in patients with PS who not have undergone any intervention. Furthermore, there was no change in risk of mortality over birth decades between men and women with PS. Diagnosis of PS within the first year of life appeared to be a specific high-risk group. However, when interpreting these data, there needs to be awareness that this study was designed to investigate patients with PS and no concomitant congenital heart lesions to avoid interference with analysis of mortality. This is in contrast to a recent study where more than 40% of the patients with repaired PS had concomitant lesions, which were predominantly atrial septal defects and persistent ductus arteriosus.^10^ Moreover, compared with other patient cohort studies, few (12.8%) patients underwent any valve intervention.

Isolated PS, if mild to moderate, is generally thought to have a good prognosis, even if intervention is sometimes needed.^14^ No prior study has followed a nationwide cohort of patients since birth and, an increased risk of mortality in patients with PS on a population level has not been previously reported. This finding should have an effect on decisions regarding follow-up and on information regarding the importance of life-time follow-up in accordance with the latest European Society of Cardiology Guidelines, 2020.^12^ Our study suggests that the condition of isolated PS should not be generally considered a benign lesion. Analysis of subgroups, such as an operation, the time period and sex, indicated that an increased risk of mortality appeared almost regardless of subgroup. An exception to this would appear to be those with a registered diagnosis after 1 year of age but as the majority of those were born before the coverage of the patient registry was complete, an unknown proportion of infants and children with isolated PS may have died before being registered, contributing to the seemingly benign prognosis in this group. By contrast, patients with an early diagnosis within the first year of life, accounted for four out of five of deaths in this study and appeared to be a high-risk group. Extra attention should be paid to these patients in follow-up. Diagnosis of PS early in life could indicate severe disease.

However, in our study, the mortality rate in patients with valve interventions appeared to be low. This finding suggests that these patients do not represent a higher risk population for mortality (eg, from postoperative progressive pulmonary valve regurgitation and its related complications and reinterventions), but further analysis on this possibility is beyond the scope of this study. The higher mortality rate in patients with no valve interventions compared with patients with valve interventions in our study raises some concern. High prevalence of severe congenital comorbidity among deaths is noteworthy and could reflect a less active treatment approach to PS related to this comorbidity.

In our study, there was no increased risk of mortality in women with PS compared with controls. Previous studies have shown that patients with stenotic vitiae and pulmonary regurgitation have an increased risk of mortality in pregnancy.^19 20^ This situation could increase risk in patients with unoperated PS and postsurgical pulmonary regurgitation. These studies suggested that an increased risk of PS in pregnancy had no effect on overall mortality in women.

### Limitations

Patient-specific data reflecting the severity of PS were not available in this study, which meant that we were unable to classify the severity in patients with PS. Furthermore, the diagnosis of PS has not been validated. Under-reporting of surgery and interventions might have occurred because the patient registries were not uniformly complete from the beginning of the study period, and children dying from complications of PS between 1970 and 1986 but unrecognised in the death registry will not have been captured. Furthermore, general under-reporting of patients from the first part of the study period is possible for the same reason, with a proportion of children probably diagnosed early but not registered before being referred to specialist cardiology at a later stage.

This study has a national perspective and includes all types of clinics. Both tertiary centres with likely many severe cases as well as regional and local outpatient clinics, which may include many mild cases as well. Finally, even though we used data from an extended period from an entire nation, the absolute number of deaths in the PS group was low.

## Conclusion

In this nationwide, register-based cohort study, we found that the risk of mortality in patients with PS was almost five times higher than controls. The risk of mortality in both patients with valve interventions and without valve interventions was higher than in matched controls. Patients with an early diagnosis of PS appear to have the highest risk of mortality. These findings suggest a close attention should be paid to long-term follow-up in this vulnerable group of patients.

## Data Availability

Data are available on reasonable request. All data relevant to the study are included in the article or uploaded as online supplemental information.
